# Modeling of paclitaxel biosynthesis elicitation in *Corylus avellana* cell culture using adaptive neuro-fuzzy inference system-genetic algorithm (ANFIS-GA) and multiple regression methods

**DOI:** 10.1371/journal.pone.0237478

**Published:** 2020-08-27

**Authors:** Siamak Farhadi, Mina Salehi, Ahmad Moieni, Naser Safaie, Mohammad Sadegh Sabet

**Affiliations:** 1 Department of Plant Genetics and Breeding, Faculty of Agriculture, Tarbiat Modares University, Tehran, Iran; 2 Department of Plant Pathology, Faculty of Agriculture, Tarbiat Modares University, Tehran, Iran; Lovely Professional University, INDIA

## Abstract

Paclitaxel as a microtubule-stabilizing agent is widely used for the treatment of a vast range of cancers. *Corylus avellana* cell suspension culture (CSC) is a promising strategy for paclitaxel production. Elicitation of paclitaxel biosynthesis pathway is a key approach for improving its production in cell culture. However, optimization of this process is time-consuming and costly. Modeling of paclitaxel elicitation process can be helpful to predict the optimal condition for its high production in cell culture. The objective of this study was modeling and forecasting paclitaxel biosynthesis in *C*. *avellana* cell culture responding cell extract (CE), culture filtrate (CF) and cell wall (CW) derived from endophytic fungus, either individually or combined treatment with methyl-β-cyclodextrin (MBCD), based on four input variables including concentration levels of fungal elicitors and MBCD, elicitor adding day and CSC harvesting time, using adaptive neuro-fuzzy inference system (ANFIS) and multiple regression methods. The results displayed a higher accuracy of ANFIS models (0.94–0.97) as compared to regression models (0.16–0.54). The great accordance between the predicted and observed values of paclitaxel biosynthesis for both training and testing subsets support excellent performance of developed ANFIS models. Optimization process of developed ANFIS models with genetic algorithm (GA) showed that optimal MBCD (47.65 mM) and CW (2.77% (v/v)) concentration levels, elicitor adding day (16) and CSC harvesting time (139 h and 41 min after elicitation) can lead to highest paclitaxel biosynthesis (427.92 μg l^-1^). The validation experiment showed that ANFIS-GA method can be a promising tool for selecting the optimal conditions for maximum paclitaxel biosynthesis, as a case study.

## Introduction

Plants are a rich source of active pharmaceutical components used in treatment of many diseases [1–7]. Some plant derived natural products (NP) such as aspirin have simple structure which could produce by chemosynthesis. However, chemical synthesis of some valuable plant NPs e.g. paclitaxel is difficult because of their complex structure [8]. On the other hand, the extraction of NPs from intact plant limit the commercial production of these compounds owing to low yield, environmental restrictions and extinction risk of these valuable pharmaceutical sources [9, 10]. Therefore, using biotechnological approaches particularly plant cell culture named “green cell factories” is a promising bioproduction platform to overcome these limitations and produce plant NPs on a large scale [1, 9, 11].

Paclitaxel as the most well-known anticancer drug is widely used for the treatment of a vast range of cancers [12]. *Taxus* species are the main source of this fantastic diterpene alkaloid, paclitaxel. Nevertheless, *Taxus* recalcitrant behavior under *in vitro* culture is a drawback for fast-growing *in vitro* culture establishment of these valuable species [13]. *Corylus avellana* is a promising alternative for paclitaxel production because of its advantages including easy *in vitro* cultivation and fast-growing cells, and also extensive availability [13–19]. Large scale production of secondary metabolite (SM) through plant cell culture needs to apply several strategies including high-yielding cell line, growth medium optimization, precursor feeding, elicitation, etc. [10, 20]. Amongst the available strategies for boosting the biosynthesis of SMs in plant *in vitro* cultures, the elicitation is considered as the most effective one [21, 22].

The combined treatment of abiotic and biotic elicitors in *Corylus avellana* [23] and *Taxus* [24] cell cultures highly boosted paclitaxel biosynthesis. Methyl-β-cyclodextrin (MBCD) has lately absorbed striking attention as an agent eliciting paclitaxel *in vitro* biosynthesis [25, 26]. On the other hand, our previous researches [9, 15, 27] indicated the positive influences of different elicitors derived from endophytic fungi, cell extract (CE), culture filtrate (CF) and cell wall (CW), on paclitaxel biosynthesis in *C*. *avellana* cell culture.

Several factors including elicitor concentration level, cell culture age and elicitor exposure time affect the process of paclitaxel biosynthesis elicitation in *C*. *avellana* cell culture [5, 11, 21], and optimal selection of these factors is a determinative issue for maximum biosynthesis of this valuable SM. Nevertheless, optimization of elicitation process is time-consuming and costly. Modeling of paclitaxel biosynthesis elicitation using mathematical methods can effectively identify the non-explicit relationships among mentioned factors and predict optimal conditions for maximum paclitaxel biosynthesis.

Multivariate statistical methods including multiple liner regression (MLR), stepwise regression (SR), ordinary least squares regression (OLSR), principal component regression (PCR) and partial least squares regression (PLSR) have been used to model biological process [28–31]. MLR studies the relationship between two or more independent variables and one dependent variable [32]. SR is a well-known data-mining method selecting the explanatory variables for regression model from a group of input variables [33]. OLSR, PCR, and PLSR are three methods to model dependent variable when there is a large number of independent variables which are highly correlated. OLSR is a statistical method estimating the relationship amongst independent variable(s) and dependent variable by minimizing sum of square differences among the predicted and observed values of dependent variable [34]. PCR is a regression method established on principal component analysis (PCA) [35]. PLSR, combining PCA and multiple regression, is a powerful modeling technique especially when the factors (input variables) are highly collinear [36].

Different predictive and fitting abilities of MLR (R^2^ = 0.32–0.91) [37] and SR (R^2^ = 0.17–0.89) [38] were demonstrated in pear rootstocks tissue culture. Also, SR was used to model phenolic profile of grapevine foliar wastes, and displayed different ability (R^2^ = 0.05–0.78) for predicting various phenolic compounds [28]. Additionally, great performance of OLSR was reported for predicting rainfed soybean and maize yields [39]. PCR (R^2^ = 0.30–0.49) was likewise applied to model grain yields based on soil properties [40]. Moreover, PLSR model was successfully used to predict important management goals in land application [41].

Poor non-linear predictive and fitting abilities of traditional modeling methods [18, 19, 38, 42–44] have shifted the studies to the use of data mining techniques such as artificial neural network (ANN) and neuro-fuzzy models. These models are able to identify and learn correlated patterns between input variables and corresponding target values in a complex and non-linear process.

ANN is a brain-inspired method that imitates the way that the human brain works [45]. It processes information and makes decision in systems involving vagueness and uncertainty [44, 46].

Adaptive neuro-fuzzy inference system (ANFIS), hybrid of fuzzy logic and neural network, incorporates the advantages of both methods including learning capabilities, interpretability, quick convergence, adaptability and high accuracy, having no disadvantages of them [47]. ANFIS displays excellent performance in approximation and prediction of nonlinear relationships in various fields [47–50]. The successful application of ANFIS as an effective modeling method in various fields explains why it has been a popular modeling approach for years. It is highly regretful that ANFIS has not been used to model SM biosynthesis in plant *in vitro* culture.

**Fig 1:**
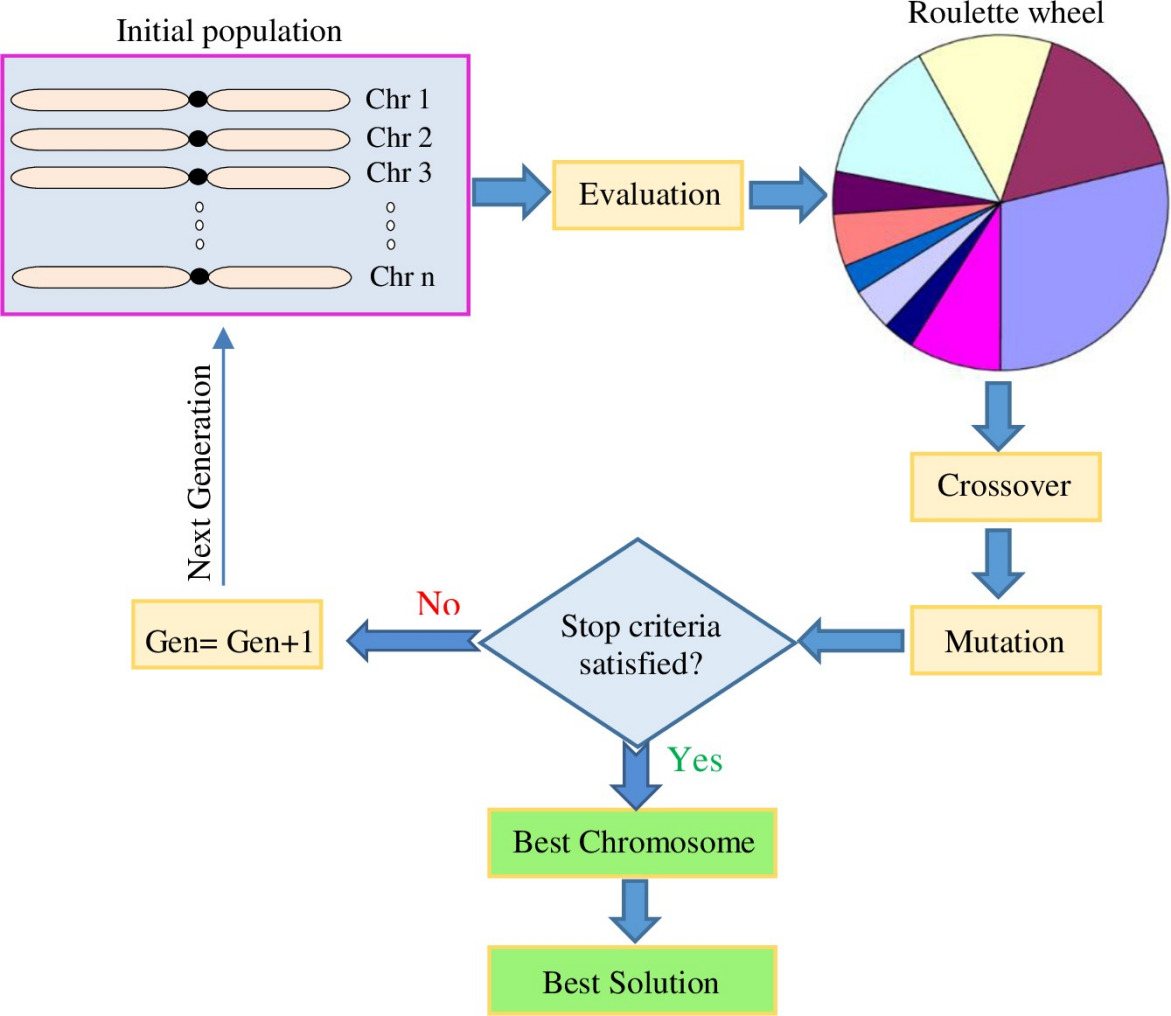
Schematic diagram of genetic algorithm.

Mathematical optimization has been successfully used in plant science [19, 49, 51–53]. Genetic algorithm (GA) as a robust optimizing tool has been successfully used to optimize culture medium composition for pear rootstock proliferation [37, 38], *in vitro* rooting of *Prunus* rootstock [54] and melon differentiation [55]. GA is the search algorithms inspired by natural selection and genetics concepts [56]. The fundamental principles of GA are the creation of an initial population of search solutions and then elite search solutions were selected for crossover using a roulette wheel selection method, which will ultimately be the best solution among them (Fig 1).

**Fig 2:**
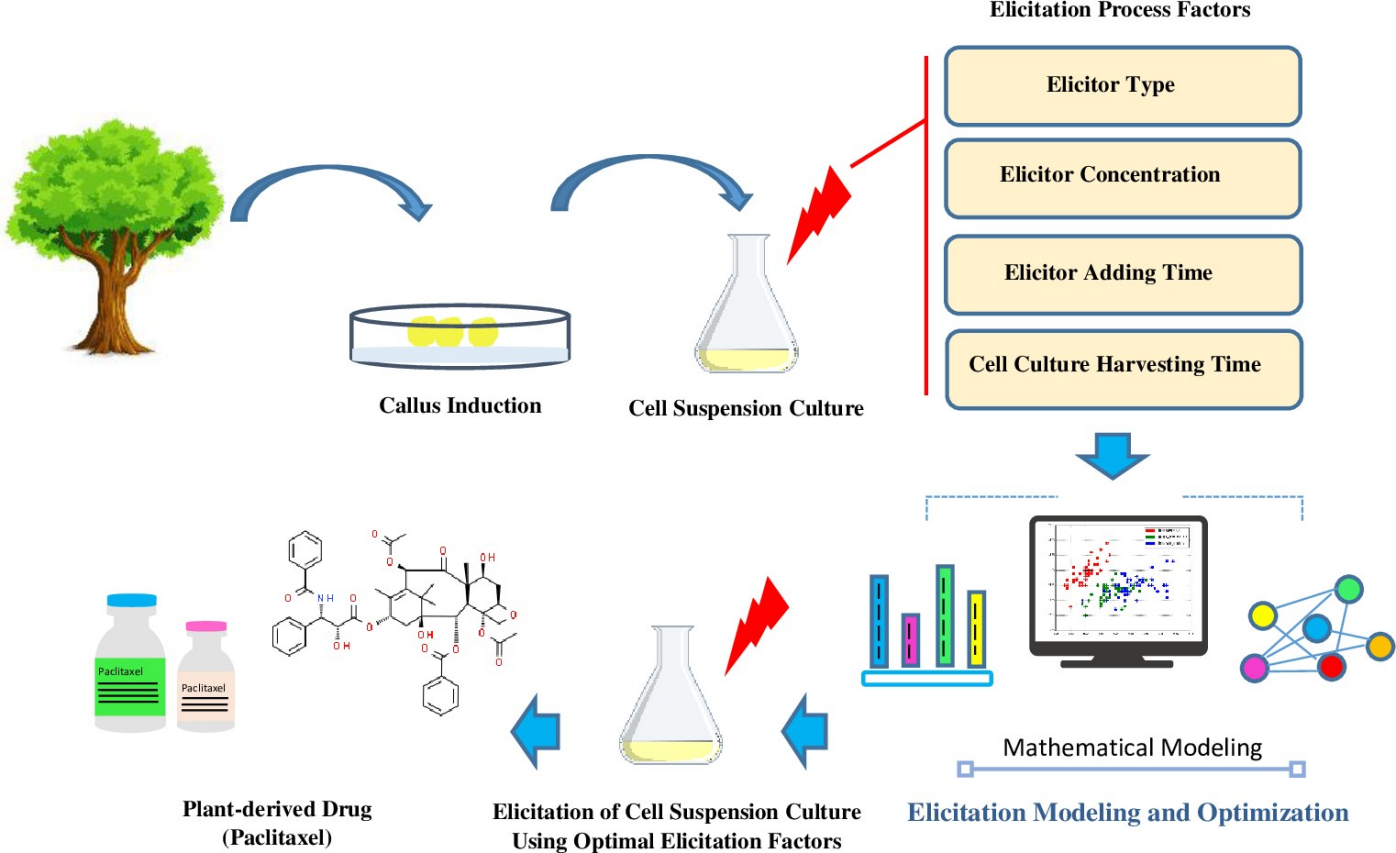
Graphical abstract of the study.

The objectives of this research were (a) to develop MLR, SR, OLSR, PCR, PLSR and ANFIS models to predict output variable “paclitaxel biosynthesis” based on input variables “concentration levels of MBCD and fungal elicitor, fungal elicitor adding day and harvesting time of cell suspension culture (CSC)” in *C*. *avellana* cell culture responding to fungal elicitors (CE, CF and CW), either individually or combined treatment with MBCD, (b) to compare performance of various mentioned models in term of prediction accuracy of paclitaxel biosynthesis, and (c) to optimize the mentioned factors for maximum paclitaxel biosynthesis by GA (Fig 2).

## Materials and methods

### Elicitation of cell suspension culture

*C*. *avellana* CSC was established as described by Salehi et al. [9, 14, 15]. Three fungal elicitors, CE, CF and CW, were used for paclitaxel biosynthesis elicitation in *C*. *avellana* CSC. Endophytic fungus applied in this research was a strain of *Coniothyrium palmarum* isolated from inner bark of *Taxus baccata*. CE, CF and CW were prepared as described previously [9, 27]. For elicitation, 1.5 ± 0.1 g of *C*. *avellana* cells (fresh mass) was cultured in 100 ml flasks containing 30 ml MS medium supplemented with 2 mg l^−1^ 2,4-D and 0.2 mg l^−1^ BAP, then treated with fungal elicitors, either individually or a combined treatment with 50 mM MBCD.

Four concentrations (1, 2.5, 5 and 10% (v/v)) of fungal elicitors including CE, CF and CW, and also mid (day 13) and late (day 17) log phase of *C*. *avellana* cell cultures were selected for adding fungal elicitors. Control received an equal volume of water (for CE)/ potato dextrose broth (PDB) (for CF)/ water containing 1% (v/v) acetic acid (for CW).

### Quantification of paclitaxel

The extraction of intracellular and extracellular paclitaxel, and also HPLC analysis were performed with a procedure described by Salehi et al. [9, 14, 15].

### Experimental design

The experiments were planned based on a randomized complete block design (RCBD) with factorial arrangement, four factors containing MBCD, fungal elicitor type, fungal elicitor concentration, fungal elicitor adding day, and three replicates. The cultures were harvested in two-day intervals after elicitation until 23^rd^ day.

### Model development

Regression and ANFIS models were individually developed for each of fungal elicitors. It is noteworthy that the models developed for each of fungal elicitors “CE, CF and CW” were designated as FCE-MOD, FCF-MOD and FCW-MOD, respectively. The data (S1 Table–S3 Table) were randomly divided into a training subset (75%) and testing one (25%), respectively. Training subset was applied to develop regression and ANFIS models, and testing subset was applied to test the predictability of developed models [57].

### Adoptive neuro-fuzzy inference system (ANFIS) model

ANFIS models were developed for each of fungal elicitors (CE, CF and CW), either individually or in a combined treatment with MBCD, to define the influences of MBCD and fungal elicitor concentration levels, fungal elicitor adding day and CSC harvesting day on paclitaxel biosynthesis.

**Fig 3:**
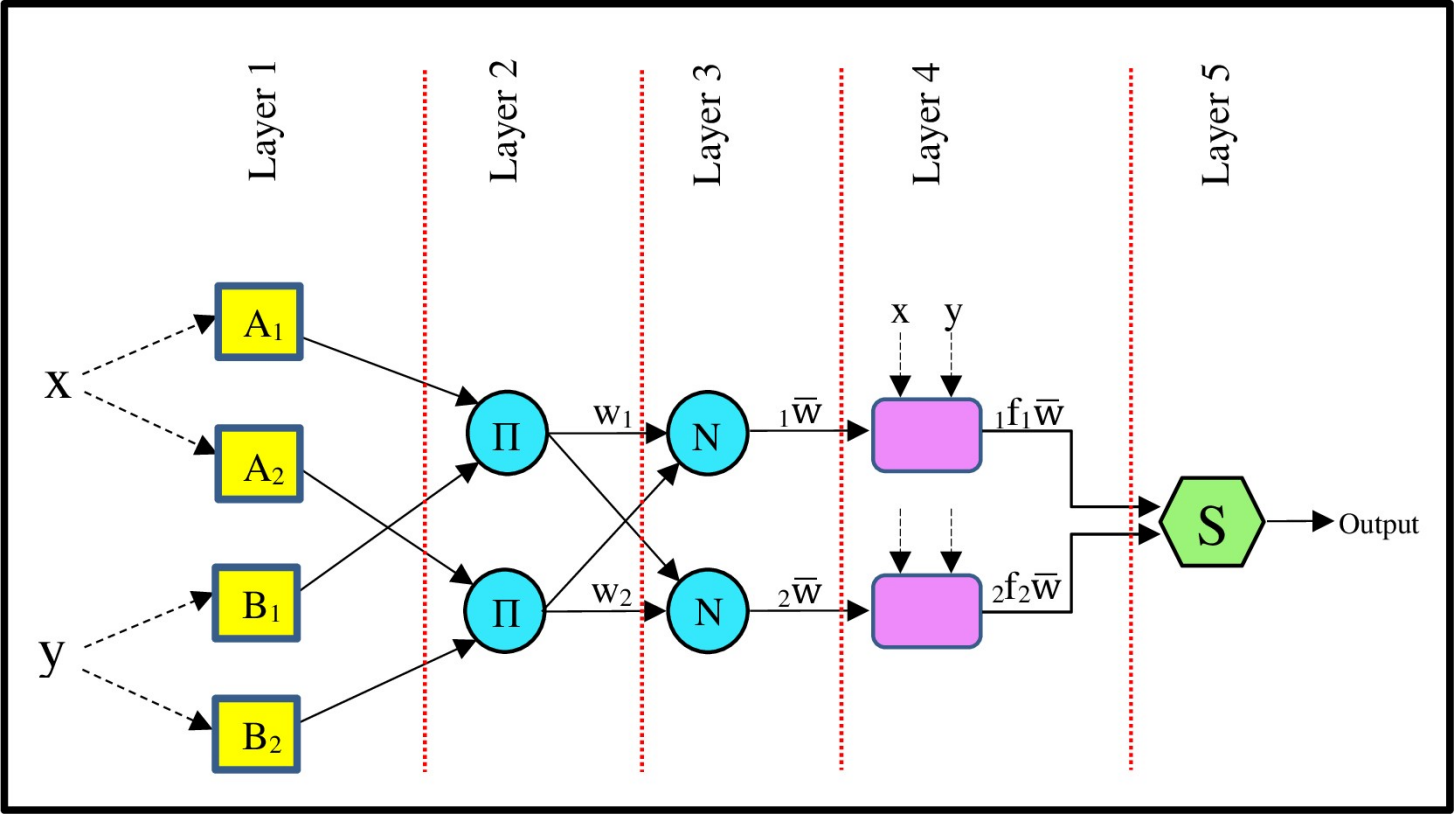
Architecture of adaptive neuro-fuzzy inference system (ANFIS) model with two input variables and one output variable.

ANFIS is made up of “if–then” rules with suitable membership functions to obtain preliminary stipulated input–output pairs (Fig 3).

Basic rule structure of ANFIS with two inputs x, y, and one output “f” can be defined as follows:

$$Rule\;1:\;if\;x\;is\;A_{1}\;and\;y\;is\;B_{1}\;then\;f_{1}=p_{1}x+q_{1}y+r_{1}$$ (1)

$$Rule\;2:\;if\;x\;is\;A_{2}\;and\;y\;is\;B_{2}\;then\;f_{2}=p_{2}x+q_{2}y+r_{2}$$ (2)

where x and y denote input variables, f is output specified by the fuzzy rules, A_i_ and B_i_ are the parameters of fuzzy sets, and p_i_, q_i_, and r_i_ are the linear parameters determined during the training process.

As shown in Fig 3, ANFIS is made up of five layers and two types of nodes, indicated by square and circle. The adaptive node (square node) accepts parameters. The fixed nodes (circle node) accept no parameter.

Layer 1 (fuzzification layer): it calculates membership values or membership degrees (μ_Ai_ and μ_Bi_) for each of input variables by membership functions. In this research, Gaussian membership function (Eq (3)) was used as membership functions. The outputs of first layer are defined by Eqs (4) and (5). {a, b, c} is called premise parameter set, and determine membership function form.

$$\mu_{A_{i}}\left(x\right)=\frac{1}{1+{\left|\frac{x-c_{i}}{a_{i}}\right|}^{2b_{i}}}$$ (3)

$$O_{1,i}=\mu_{A_{i}}\left(x\right)\;i=1,2$$ (4)

$$O_{1,i}=\mu_{B_{i-2}}\left(x\right)\;i=3,4$$ (5)

Layer 2 (rule layer): each node (Π) computes firing strengths (w_i_) (Eq (6)) for fuzzy rules via the multiplication of input signals of corresponding fuzzification nodes.

$$O_{2,i}=w_{i}=\mu_{A_{i}}\left(x\right).\mu_{B_{i}}\left(y\right),\;i=1,2$$ (6)

Layer 3 (normalisation layer): each node (N) computes the ratio of its own rule firing strength to sum of all rules’s firing strengths, normalized firing strengths, as given in Eq (7).

$$O_{3,i}={\bar{w}}_{i}=\frac{w_{i}}{w_{1}+w_{2}}\;i=1,2$$ (7)

Layer 4 (defuzzification layer): each node computes weighted values of rules by the multiplication of normalized firing strength (${\bar{w}}_{i}$) and consequent function as given in Eq (8). Consequent function is first order polynomial of consequence parameters {pi, qi, ri}.

$$O_{4,i}={\bar{w}}_{i}z_{i}={\bar{w}}_{i}\left(p_{x}+q_{i}y+r_{i}\right)\;i=1,2$$ (8)

Layer 5 (summation layer): This single-node layer (S) computes final output by the summation of all incoming signals from all defuzzification layer nodes as follows:

$$O_{5,i}=\underset{i}{\sum }{\bar{w}}_{i}f_{i}=\frac{\sum_{i}w_{i}f_{i}}{\sum_{i}w_{i}}\;i=1,2$$ (9)

Hybrid learning rules, back-propagation algorithm and least-squares estimate [49, 58] were used to tune premise parameter set and consequent parameters.

The performance of ANFIS models is determined by three statistical criteria including root mean square error (RMSE) (Eq (10)), mean absolute error (MAE) (Eq (11)) and coefficient of determination (R^2^) (Eq (12)).

$$RMSE=\sqrt{\left(\overset{n}{\underset{i=1}{\sum }}{\left(y_{est}-y_{act}\right)}^{2}\right)/n}$$ (10)

$$MAE=1/n\sum_{i=1}^{n}\left|y_{est}-y_{act}\right|$$ (11)

$$R^{2}=1-\left(\overset{n}{\underset{i=1}{\sum }}{\left(y_{est}-y_{act}\right)}^{2}/\overset{n}{\underset{i=1}{\sum }}{\left(y_{act}-\bar{y}\right)}^{2}\right)$$ (12)

Where “y_act_” are the actual values, “y_est_” are the predicted values, and “n” is the number of data.

### Optimization process by genetic algorithm (GA)

GA was applied to optimize the value of input variables (MBCD and fungal elicitor concentration levels, fungal elicitor adding day and CSC harvesting time) in developed ANFIS models for maximum paclitaxel biosynthesis. An initial population of 200, crossover rate of 0.7, generation number of 1000, mutation rate of 0.03 and uniform function as a mutation function, two-point crossover function, and a roulette wheel selection function were set to select the optimal levels of input variables (Fig 1).

### Sensitivity analysis of the models

The sensitivity of paclitaxel biosynthesis against input variables (MBCD and fungal elicitor concentration levels, fungal elicitor adding day and CSC harvesting time) was determined by the criteria including variable sensitivity error (VSE) value displaying the performance (RMSE) of ANFIS model when that particular input variable is unavailable in the model. Variable sensitivity ratio (VSR) value was calculated as ratio of VSE and ANFIS model error (RMSE value) when all input variables are available. The input variable with higher VSR was considered as higher important variable in model.

The mathematical codes for the development and evaluation of ANFIS and regression models were written using MATLAB software [59] and XLSTAT [60], respectively, and the graphs were made by GraphPad Prism 5 [61] software.

### Validation experiment

CE, CF, CW and MBCD concentration levels, fungal elicitor adding day, and CSC harvesting time optimized by ANFIS-GA were examined to evaluate the efficiency of ANFIS-GA model for forecasting and optimizing paclitaxel biosynthesis in *C*. *avellana* cell culture responding to used elicitors. The culture conditions for *C*. *avellana* cell growth remained the same as mentioned above.

## Results and discussion

### Modelling of paclitaxel biosynthesis using regression and ANFIS models

Elicitation is the most important and promising approach for increasing SMs biosynthesis in plant cell culture platform. However optimization of elicitation process is a key step for achieving this goal. The type, concentration level and adding day of elicitors, and also CSC harvesting time are effective factors in paclitaxel biosynthesis in *C*. *avellana* CSC elicited by different elicitors [9, 15, 27]. Predicting the optimal amount of these mentioned factors is highly promising and essential for paclitaxel biosynthesis increment and cost decrement.

To model paclitaxel biosynthesis in *C*. *avellana* cell culture responding to fungal elicitor and MBCD by regression and ANFIS methods, fungal elicitor and MBCD concentration levels, fungal elicitor adding day and CSC harvesting time were used as input variables, and paclitaxel biosynthesis as output variable. Here, various regression methods (MLR, SR, OLSR, PCR and PLSR) were tested to find the best regression model to predict paclitaxel biosynthesis in *C*. *avellana* responding to fungal elicitors and MBCD. All developed regression models for three elicitors “CE, CF and CW” showed statistically significant relationships between output variable “paclitaxel biosynthesis” and input variables (fungal elicitor and MBCD concentration levels, fungal elicitor adding day and CSC harvesting day),” (Table 1). MLR, SR, OLSR, PCR and PLSR models developed for paclitaxel biosynthesis regarding fungal elicitor and MBCD concentration levels, fungal elicitor adding day and CSC harvesting time were shown in Table 1. Goodness-of-fit of MLR, SR, OLSR, PCR and PLSR models was performed to detect the best model for predicting paclitaxel biosynthesis. High significant R^2^ value and low RMSE and MAE values displayed the model capability.

**Table 1:** Statistics of multiple linear regression (MLR), stepwise regression (SR), ordinary least squares regression (OLSR), principal component regression (PCR), partial least squares regression (PLSR) and adaptive neuro-fuzzy inference system (ANFIS) for paclitaxel biosynthesis modeling in *Corylus avellana* cell culture exposed to different concentration of fungal cell extract (FCE-MOD), fungal culture filtrate (FCF-MOD) and fungal cell wall (FCW-MOD) elicitors, either individually or combined treatment with 50 mM methyl-β-cyclodextrin (MBCD).

	Models	Training subsets			Testing subsets			Pr > F
		R^2^	RMSE	MAE	R^2^	RMSE	MAE	
**FCE-MOD**	**MLR**	0.17	48.29	36.48	0.19	41.13	33.41	< 0.0001
	**SR**	0.17	48.27	36.96	0.20	40.93	33.50	< 0.0001
	**PLSR**	0.16	47.81	36.82	0.20	40.89	33.57	< 0.0001
	**PCR**	0.17	48.29	36.21	0.19	41.14	33.46	< 0.0001
	**OLSR**	0.17	48.29	36.21	0.19	41.14	33.46	< 0.0001
	**ANFIS**	**0.94**	**12.60**	**9.69**	**0.88**	**16.68**	**12.57**	< 0.0001
**FCF-MOD**	**MLR**	0.54	37.86	29.07	0.61	32.53	26.35	< 0.0001
	**SR**	0.54	37.78	24.32	0.61	32.57	26.23	< 0.0001
	**PLSR**	0.53	37.50	29.00	0.61	32.50	26.67	< 0.0001
	**PCR**	0.54	37.86	28.81	0.61	32.54	26.35	< 0.0001
	**OLSR**	0.54	37.86	28.81	0.61	32.54	26.35	< 0.0001
	**ANFIS**	**0.95**	**11.87**	**8.41**	**0.90**	**16.18**	**10.55**	< 0.0001
**FCW-MOD**	**MLR**	0.37	71.50	72.01	0.30	71.94	54.64	< 0.0001
	**SR**	0.37	71.32	55.51	0.30	71.98	54.29	< 0.0001
	**PLSR**	0.36	71.45	52.62	0.33	69.15	51.81	< 0.0001
	**PCR**	0.38	71.50	52.81	0.30	71.92	54.61	< 0.0001
	**OLSR**	0.38	71.50	52.81	0.30	71.92	54.61	< 0.0001
	**ANFIS**	**0.97**	**14.04**	**2.35**	**0.94**	**20.19**	**13.77**	< 0.0001

R^2^: coefficient of determination, RMSE: root mean square error, MAE: mean absolute error.

**Fig 4:**
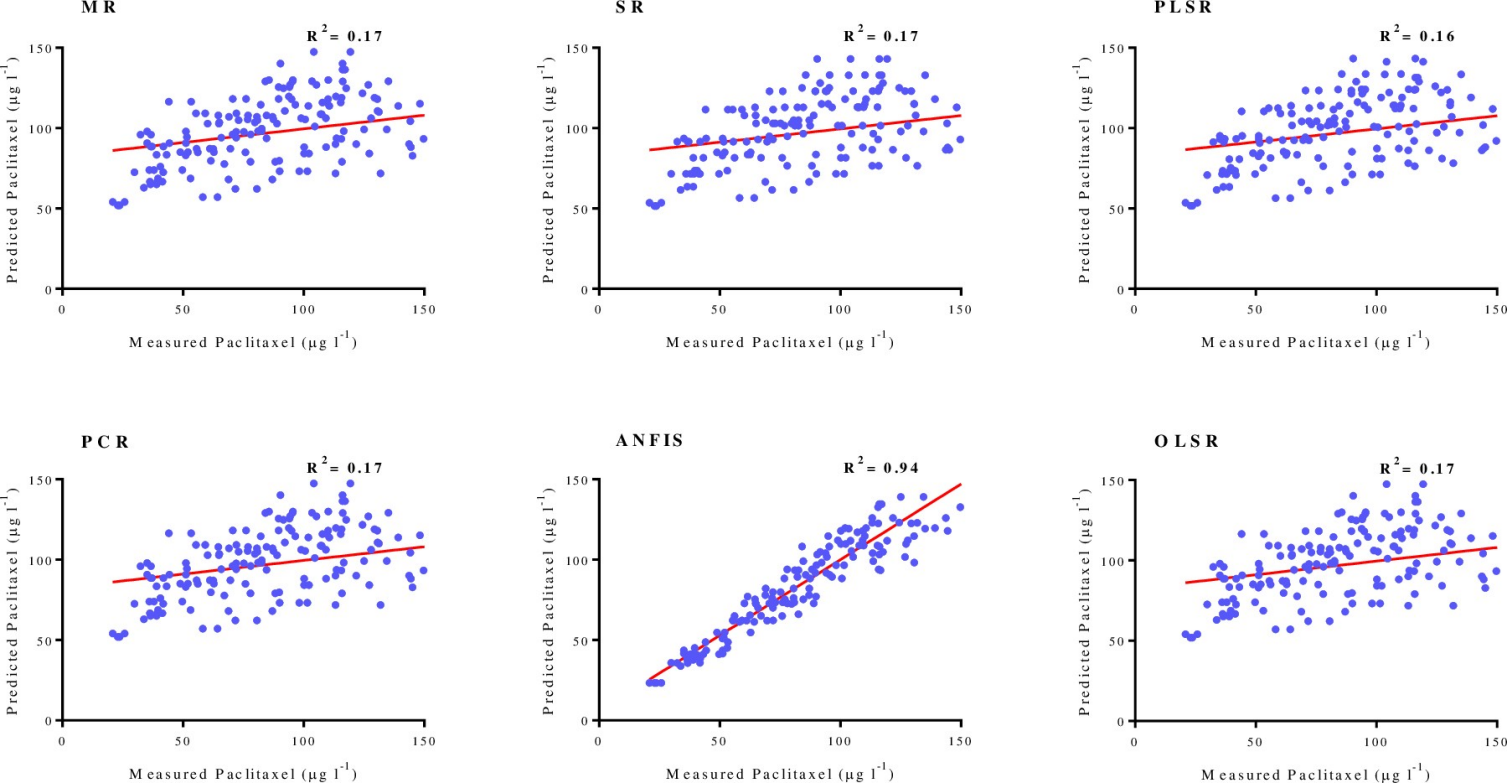
Scatter plot of actual data against predicted values of paclitaxel biosynthesis in *Corylus avellana* cell cultures exposed with different concentration of fungal cell extract (CE), either individually or combined treatment with 50 mM methyl-β-cyclodextrin (MBCD), using adaptive neuro-fuzzy inference system (ANFIS), multiple liner regression (MLR) stepwise regression (SR), ordinary least squares regression (OLSR), principal component regression (PCR) and partial least squares regression (PLSR) models in training subset. The solid line shows fitted simple regression line on scatter points.

**Fig 5:**
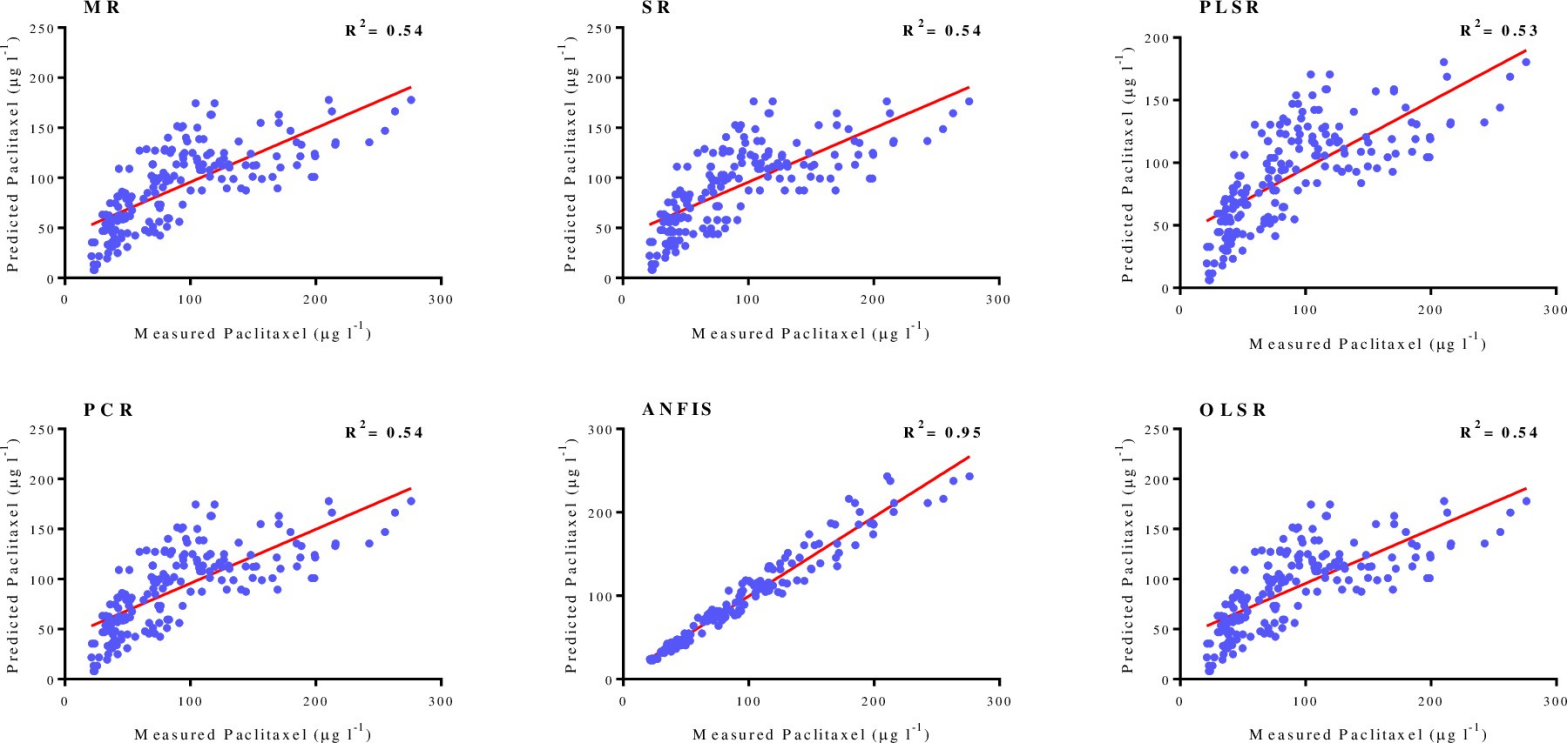
Scatter plot of actual data against predicted values of paclitaxel biosynthesis in *Corylus avellana* cell cultures exposed with different concentration of fungal culture filtrate (CF), either individually or combined treatment with 50 mM methyl-β-cyclodextrin (MBCD), using adaptive neuro-fuzzy inference system (ANFIS), multiple liner regression (MLR) stepwise regression (SR), ordinary least squares regression (OLSR), principal component regression (PCR) and partial least squares regression (PLSR) models in training subset. The solid line shows fitted simple regression line on scatter points.

**Fig 6:**
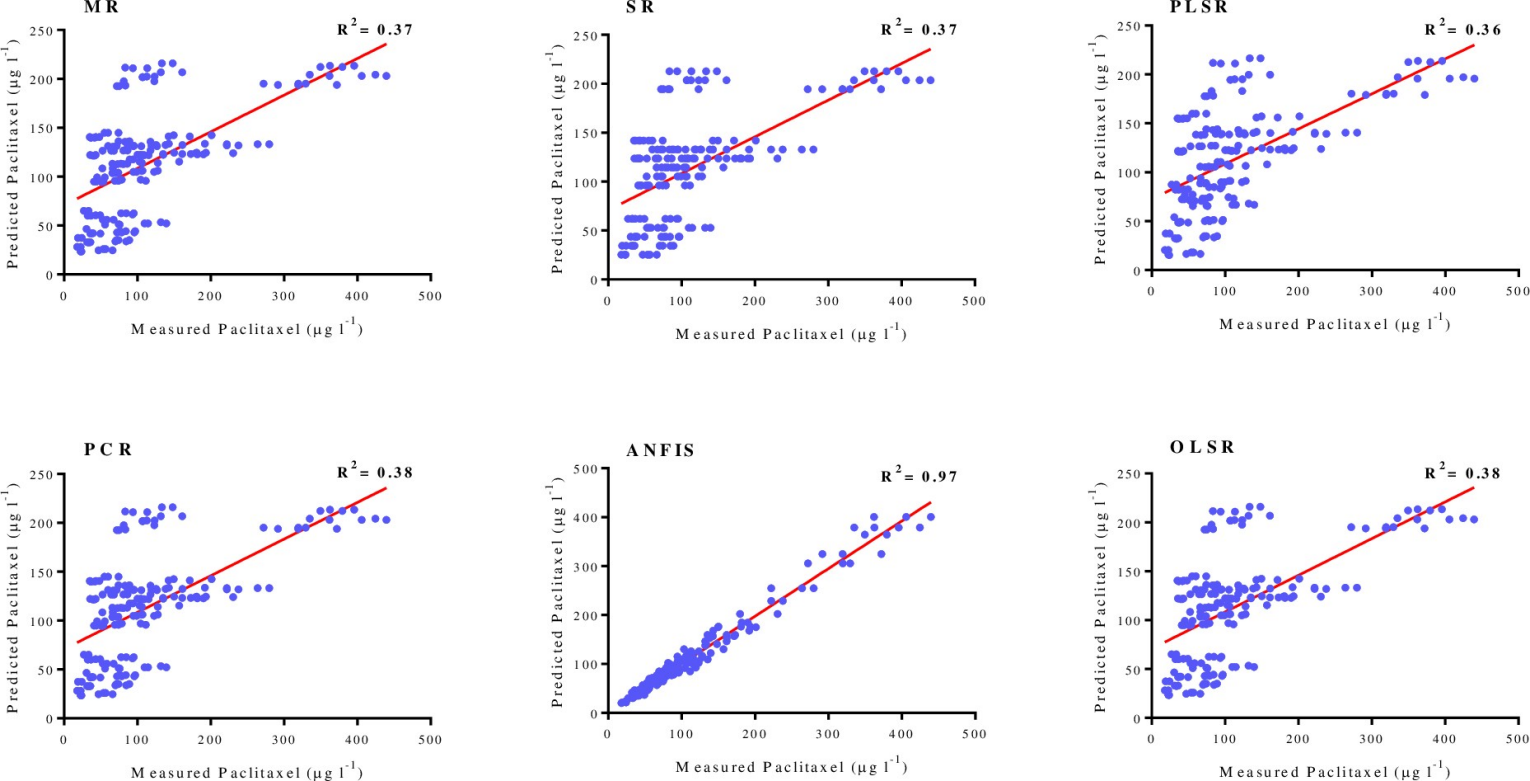
Scatter plot of actual data against predicted values of paclitaxel biosynthesis in *Corylus avellana* cell cultures exposed with different concentration of fungal cell wall (CW), either individually or combined treatment with 50 mM methyl-β-cyclodextrin (MBCD), using adaptive neuro-fuzzy inference system (ANFIS), multiple liner regression (MLR) stepwise regression (SR), ordinary least squares regression (OLSR), principal component regression (PCR) and partial least squares regression (PLSR) models in training subset. The solid line shows fitted simple regression line on scatter points.

As shown in Table 1, goodness-of-fit displayed no difference regarding the accuracy of regression models for paclitaxel biosynthesis for training and testing subsets in FCF-, FCE- and FCW-MOD. The performance of developed MLR, SR, OLSR, PCR and PLSR models were evaluated by plotting the predicted values against the observed values of training subset (FCE-MOD: R^2^ = 0.17, 0.17, 0.16, 0.17 and 0.17; FCE-MOD: R^2^ = 0.54, 0.54, 0.53, 0.54 and 0.54; FCE-MOD: R^2^ = 0.37, 0.37, 0.36, 0.38 and 0.38, respectively) (Figs 4–6). R^2^ values for testing subset suggested the best mentioned models can explain 17, 54 and 38% variability in paclitaxel biosynthesis in FCE-MOD, FCF-MOD and FCW-MOD, respectively, when they face with unseen data (Table 1).

Paclitaxel biosynthesis was predicted according to developed ANFIS model. To evaluate the performance of developed ANFIS models, the predicted values were plotted against the observed values of training subset (FCE-MOD: R^2^ = 0.94; FCF-MOD: R^2^ = 0.95; FCW-MOD: R^2^ = 0.97) (Figs 4–6). The great accordance between the predicted and observed values of paclitaxel was observed for both training and testing subsets (Table 1). Goodness-of-fit of developed ANFIS models showed that the developed models could accurately predict paclitaxel biosynthesis of testing subset (0.88, 0.90 and 0.94 in FCE-MOD, FCF-MOD and FCW-MOD, respectively), not used during training processes (Table 1). Also, developed ANFIS models displayed the balanced statistical values for both training and testing subsets (Table 1). The statistical values for ANFIS models displayed the higher prediction accuracy as compared to regression models, as estimated R^2^ for ANFIS vs. best regression models were 0.94 vs. 0.17 in FCE-MOD; 0.95 vs. 0.54 in FCF-MOD and 0.97 vs. 0.38 in FCW-MOD (Table 1). Comparing ANFIS and MLR, SR, PLSR, PCR and OLSR models performed regarding prediction accuracy displayed a higher accuracy of ANFIS models as compared to regression models (Table 1). The superiority of artificial intelligence (AI) models than regression method was demonstrated in culture medium formulation for pear rootstock proliferation [37, 38]. Also, AI displayed a higher accuracy as compared to regression method in predicting targeted phenolic profile of grapevine foliar wastes [28].

Our results suggested that ANFIS models could accurately predict paclitaxel biosynthesis in *C*. *avellana* CSC (Table 1). Very small absolute error values (Table 1) showed the high potential of ANFIS models in predicting output variable, paclitaxel biosynthesis.

Paclitaxel biosynthesis is the complex biological processes which require the accurate techniques for modeling and optimization. As observed in this study, AI technology has exhibited high powerful potential for modeling the complex relationships in biological systems, and displaying the superior prediction performances as compared to classical statistics [19, 28, 44].

### Sensitivity analysis of the developed models

Regardless of previous studies on the effects of CE and CF concentration levels, fungal elicitor adding day and CSC harvesting time on paclitaxel biosynthesis, there remains the question to be answered; which input variables are the most important in paclitaxel biosynthesis. Sensitivity analysis determines the role of input variables in ANFIS models. Indeed, sensitivity analysis shed light on relationships between “MBCD and fungal elicitor concentration levels, fungal elicitor adding day and CSC harvesting time” and paclitaxel biosynthesis. To rank input variables based on their relative importance in the model, VSRs were estimated. VSRs were obtained for paclitaxel biosynthesis regarding fungal elicitor (CE, CF and CW) and MBCD concentration levels, fungal elicitor adding day and CSC harvesting time (Table 2). Analysis of FCE-MOD indicated that paclitaxel biosynthesis was more sensitive to CSC harvesting time (VSR = 3.32), followed by fungal elicitor (CE) concentration level (VSR = 2.41), fungal elicitor adding day (VSR = 2.24) and MBCD concentration level (VSR = 1.89) (Table 2). According to analysis of FCF-MOD, paclitaxel biosynthesis displayed more sensitivity to CSC harvesting time (VSR = 2.62), followed by fungal elicitor (CF) concentration level (VSR = 1.88), fungal elicitor adding day (VSR = 1.28) and MBCD concentration level (VSR = 1.05). Accordingly, paclitaxel biosynthesis in FCW-MOD exhibited more sensitivity to CSC harvesting time (VSR = 2.43), followed by fungal elicitor (CW) concentration level (VSR = 2.04), fungal elicitor adding day (VSR = 1.42) and MBCD concentration level (VSR = 0.98) (Table 2). Overall, sensitivity analysis displayed that CSC harvesting time and fungal elicitor concentration level are the most important variables affecting paclitaxel biosynthesis (Table 2).

**Table 2:** Importance (according to the sensitivity analysis) and optimal levels of the different factors including fungal cell extract (CE; FCE-MOD), culture filtrate (CF; FCF-MOD) and cell wall (CW; FCW-MOD) concentration level, methyl-β-cyclodextrin (MBCD) concentration level, elicitor adding day and harvesting time (day) for achieving maximum paclitaxel biosynthesis in *Corylus avellana* cell suspension culture (CSC) using adaptive neuro-fuzzy inference system-genetic algorithm (ANFIS-GA).

	Variable	Importance value (according to VSR^a^)	Optimal level	Optimal output
**FCE-MOD**	CE concentration level (% (v/v))	2.41	4.30	294.35
	MBCD concentration (mM)	1.89	2.00	
	Elicitor Adding day	2.24	16	
	CSC Harvest time	3.32	20.12	
**FCF-MOD**	CF concentration level (% (v/v))	1.88	8.4	271.77
	MBCD concentration (mM)	1.05	42.35	
	Elicitor Adding day	1.28	16.75	
	CSC Harvest time	2.62	22	
**FCW-MOD**	CW concentration level (% (v/v))	2.04	2.77	427.92
	MBCD concentration (mM)	0.98	47.65	
	Elicitor Adding day	1.42	16	
	CSC Harvest time	2.43	21.82	

^a^ Relative indication of the ratio between the variable sensitivity error and the error of the model when all variables are available.

### Model optimization and validation experiment

GA has been efficiently used to solve problems with extremely difficult and unknown solution in various fields [19, 38, 54, 62]. The optimization analysis on developed ANFIS models was performed using GA to determine optimal levels of input variables for achieving maximum paclitaxel biosynthesis in *C*. *avellana* CSCs (Table 2). The optimization results of paclitaxel biosynthesis in FCE-MOD showed that adding 4.30% (v/v) of *C*. *palmarum* CE on 16^th^ day to cell culture pre-treated with 2 mM MBCD, and harvesting CSC 98 h and 53 min after elicitation could result in the maximum paclitaxel content (294.35 μg l^-1^) (Table 2). Also, optimization results in FCF-MOD indicated that the highest content of paclitaxel (271.77 μg l^-1^) may produce by adding 8.4% (v/v) CF on 17^th^ day to cell culture pre-treated with 42.35 mM MBCD and harvesting CSC 126 h after elicitation (Table 2). Accordingly, the optimal levels of input variables for achieving maximum paclitaxel biosynthesis (427.92 μg l^-1^) in FCW-MOD are adding 2.77% (v/v) CW on 16^th^ day to cell culture pre-treated with 47.65 mM MBCD and harvesting CSC 139 h and 41 min after elicitation (Table 2).

To test the efficiency of ANFIS-GA models for forecasting and optimizing paclitaxel biosynthesis in *C*. *avellana* CSC responding to fungal elicitors and MBCD, *C*. *avellana* cell culture exposed to optimized input variables in ANFIS models using GA. *C*. *avellana* cell culture pre-treated with 2 mM MBCD exposed to 4.30% (v/v) CE on 16^th^ day, and harvesting it 98 h and 53 min after elicitation, produced 278.12 ± 23.64 μg l^-1^ paclitaxel. Also, adding 8.4% (v/v) CF on 17^th^ day to *C*. *avellana* cell culture pre-treated with 42.35 mM MBCD and harvesting CSC 126 h after elicitation resulted in paclitaxel biosynthesis of 283.83 ± 21.39 μg l^-1^. Validation experiment of FCW-MOD showed that *C*. *avellana* cell culture exposed to optimized input variables by GA (CW concentration: 2.77% (v/v); MBCD concentration: 47.65 mM; CW adding day: 16^th^ day; CSC harvesting time: 139 h and 41 min after elicitation) biosynthesized 402.92 ± 34.48 μg l^-1^ paclitaxel (Table 3). These results show validity of developed ANFIS-GA for forecasting and optimizing paclitaxel biosynthesis in *C*. *avellana* CSC responding fungal elicitors and MBCD. This is the first study on predicting the optimal conditions for maximum paclitaxel biosynthesis in *C*. *avellana* CSC exposed to fungal elicitors in combine with MBCD using ANFIS-GA.

**Table 3:** Paclitaxel biosynthesis in *Corylus avellana* cell suspension culture (CSC) exposed to optimized fungal elicitor and methyl-β–cyclodextrin concentration levels, fungal elicitor adding day and CSC harvesting time in adaptive neuro-fuzzy inference system (ANFIS) models using genetic algorithm (GA), and predicted paclitaxel biosynthesis via ANFIS-GA.

Treatment	Paclitaxel (μg l^-1^)		
	FCE-MOD	FCF-MOD	FCW-MOD
Predicted via ANFIS-GA	294.35	271.77	427.92
Tested in validation experiment	278.12 ± 23.64	283.83 ± 21.39	402.92 ± 34.48

FCE-MOD: fungal cell extract model, FCF-MOD: fungal culture filtrate model, FCW-MOD: fungal cell wall model.

## Conclusion

This research applied regression and ANFIS-GA models for forecasting and optimizing paclitaxel biosynthesis in *C*. *avellana* cell culture treated with fungal elicitor and MBCD for the first time. The great accordance between the predicted and observed values of paclitaxel biosynthesis supports excellent performance of developed ANFIS models for modeling paclitaxel biosynthesis. Overall, AI models like ANFIS, effectively handle complex input–output patterns and display a supreme ability for modeling and forecasting results, and present an effective guidance for improving the biosynthesis of SMs in plant *in vitro* culture. Since this research focused on ANFIS as one of AI method for modeling paclitaxel biosynthesis in *C*. *avellana* cell culture, as a case study, it is recommended to evaluate other AI methods to model SM biosynthesis in plant *in vitro* culture in future studies.

## Supporting information

S1 Table Levels of input variables related to paclitaxel biosynthesis in *Corylus avellana* cell culture responding to cell extract (CE) and methyl-β-cyclodextrin (MBCD). (DOCX)

S2 Table Levels of input variables related to paclitaxel biosynthesis in *Corylus avellana* cell culture responding to culture filtrate (CF) and methyl-β-cyclodextrin (MBCD). (DOCX)

S3 Table Levels of input variables related to paclitaxel biosynthesis in *Corylus avellana* cell culture responding to cell wall (CW) and methyl-β-cyclodextrin (MBCD). (DOCX)

## References

[1] Karuppusamy S. A review on trends in production of secondary metabolites from higher plants by in vitro tissue, organ and cell cultures. Journal of Medicinal Plants Research. 2009; 3(13):1222–39.

[2] Li JW-H, Vederas JC. Drug discovery and natural products: end of an era or an endless frontier? Science. 2009; 325(5937):161–5. 10.1126/science.1168243 19589993

[3] Newman DJ, Cragg GM. Natural products as sources of new drugs over the 30 years from 1981 to 2010. Journal of natural products. 2012; 75(3):311–35. 10.1021/np200906s 22316239 PMC3721181

[4] Salehi M, Naghavi MR, Bahmankar M. A review of *Ferula* species: Biochemical characteristics, pharmaceutical and industrial applications, and suggestions for biotechnologists. Industrial Crops and Products. 2019; 139:111511.

[5] Salehi M, Karimzadeh G, Naghavi MR. Synergistic effect of coronatine and sorbitol on artemisinin production in cell suspension culture of *Artemisia annua* L. cv. Anamed. Plant Cell, Tissue and Organ Culture (PCTOC). 2019; 137(3):587–97.

[6] Salehi M, Karimzadeh G, Naghavi MR, Badi HN, Monfared SR. Expression of key genes affecting artemisinin content in five *Artemisia* species. Scientific reports. 2018; 8(1):1–11. 10.1038/s41598-017-17765-5 30139985 PMC6107673

[7] Salehi M, Karimzadeh G, Naghavi MR, Badi HN, Monfared SR. Expression of artemisinin biosynthesis and trichome formation genes in five *Artemisia* species. Industrial crops and products. 2018; 112:130–40.

[8] De Luca V, Salim V, Atsumi SM, Yu F. Mining the biodiversity of plants: a revolution in the making. Science. 2012; 336(6089):1658–61. 10.1126/science.1217410 22745417

[9] Salehi M, Moieni A, Safaie N, Farhadi S. Elicitors derived from endophytic fungi *Chaetomium globosum* and *Paraconiothyrium brasiliense* enhance paclitaxel production in *Corylus avellana* cell suspension culture. Plant Cell, Tissue and Organ Culture (PCTOC). 2019; 136(1):161–71.

[10] Ochoa-Villarreal M, Howat S, Hong S, Jang MO, Jin Y-W, Lee E-K, et al Plant cell culture strategies for the production of natural products. BMB reports. 2016; 49(3):149 10.5483/bmbrep.2016.49.3.264 26698871 PMC4915229

[11] Marchev AS, Yordanova ZP, Georgiev MI. Green (cell) factories for advanced production of plant secondary metabolites. Critical Reviews in Biotechnology. 2020; 40(4):443–58. 10.1080/07388551.2020.1731414 32178548

[12] Wani MC, Taylor HL, Wall ME, Coggon P, McPhail AT. Plant antitumor agents. VI. Isolation and structure of taxol, a novel antileukemic and antitumor agent from *Taxus brevifolia*. Journal of the American Chemical Society. 1971; 93(9):2325–7. 10.1021/ja00738a045 5553076

[13] Bestoso F, Ottaggio L, Armirotti A, Balbi A, Damonte G, Degan P, et al In vitro cell cultures obtained from different explants of *Corylus avellana* produce Taxol and taxanes. BMC biotechnology. 2006; 6(1):45. 17150090 10.1186/1472-6750-6-45 PMC1702537

[14] Salehi M, Moieni A, Safaie N. A novel medium for enhancing callus growth of hazel (*Corylus avellana* L.). Scientific reports. 2017; 7(1):1–9. 10.1038/s41598-016-0028-x 29142273 PMC5688170

[15] Salehi M, Moieni A, Safaie N, Farhadi S. New synergistic co-culture of *Corylus avellana* cells and *Epicoccum nigrum* for paclitaxel production. Journal of industrial microbiology & biotechnology. 2019; 46(5):613–23. 30783891 10.1007/s10295-019-02148-8

[16] Service RF. Hazel trees offer new source of cancer drug. Science (New York, NY). 2000; 288(5463):27. 10.1126/science.288.5463.27a 10766629

[17] Salehi M, Moieni A, Safaie N. Elicitors derived from hazel (*Corylus avellana* L.) cell suspension culture enhance growth and paclitaxel production of *Epicoccum nigrum*. Scientific reports. 2018; 8(1):1–10. 10.1038/s41598-017-17765-5 30104672 PMC6089963

[18] Salehi M, Moieni A, Safaie N, Farhadi S. Whole fungal elicitors boost paclitaxel biosynthesis induction in *Corylus avellana* cell culture. PLoS ONE. 2020; 15(7): e0236191. 10.1371/journal.pone.0236191 PMC7365444 32673365

[19] Salehi M, Farhadi S, Moieni A, Safaie N, Ahmadi H. Mathematical modeling of growth and paclitaxel biosynthesis in *Corylus avellana* cell culture responding to fungal elicitors using multilayer perceptron-genetic algorithm. Frontiers in plant science. 2020; 10.3389/fpls.2020.01148 PMC7432144 32849706

[20] Bonfill M, Exposito O, Moyano E, Cusidó R, Palazón J, Pinol M. Manipulation by culture mixing and elicitation of paclitaxel and baccatin III production in *Taxus baccata* suspension cultures. In Vitro Cellular & Developmental Biology-Plant. 2006; 42(5):422–6.

[21] Zhong J-J. Plant cell culture for production of paclitaxel and other taxanes. Journal of Bioscience and Bioengineering. 2002; 94(6):591–9. 10.1016/s1389-1723(02)80200-6 16233355

[22] Wang W, Zhong J-J. Manipulation of ginsenoside heterogeneity in cell cultures of *Panax notoginseng* by addition of jasmonates. Journal of bioscience and bioengineering. 2002; 93(1):48–53. 16233164

[23] Rezaei A, Ghanati F, Behmanesh M, Mokhtari-Dizaji M. Ultrasound-potentiated salicylic acid–induced physiological effects and production of taxol in hazelnut (*Corylus avellana* L.) cell culture. Ultrasound in medicine & biology. 2011; 37(11):1938–47. 21835541 10.1016/j.ultrasmedbio.2011.06.013

[24] Zhang C, Mei X, Liu L, Yu L. Enhanced paclitaxel production induced by the combination of elicitors in cell suspension cultures of *Taxus chinensis*. Biotechnology Letters. 2000; 22(19):1561–4.

[25] Ramirez-Estrada K, Osuna L, Moyano E, Bonfill M, Tapia N, Cusido RM, et al Changes in gene transcription and taxane production in elicited cell cultures of *Taxus× media* and *Taxus globosa*. Phytochemistry. 2015; 117:174–84. 10.1016/j.phytochem.2015.06.013 26091963

[26] Sabater‐Jara AB, Onrubia M, Moyano E, Bonfill M, Palazón J, Pedreño MA, et al Synergistic effect of cyclodextrins and methyl jasmonate on taxane production in *Taxus x media* cell cultures. Plant biotechnology journal. 2014; 12(8):1075–84. 10.1111/pbi.12214 24909837

[27] Farhadi S, Moieni A, Safaie N, Sabet MS, Salehi M. Fungal cell wall and methyl-β–cyclodextrin synergistically enhance paclitaxel biosynthesis and secretion in *Corylus avellana* cell suspension culture. Scientific reports. 2020; 10(1):1–10. 10.1038/s41598-019-56847-4 32214149 PMC7096423

[28] Eftekhari M, Yadollahi A, Ahmadi H, Shojaeiyan A, Ayyari M. Development of an artificial neural network as a tool for predicting the targeted phenolic profile of grapevine (*Vitis vinifera*) foliar wastes. Frontiers in plant science. 2018; 9:837 10.3389/fpls.2018.00837 29971086 PMC6018394

[29] Du C, Wei J, Wang S, Jia Z. Genomic selection using principal component regression. Heredity. 2018; 121(1):12–23. 10.1038/s41437-018-0078-x 29713089 PMC5997717

[30] Costa C, Menesatti P, Spinelli R. Performance modelling in forest operations through partial least square regression. Silva Fennica. 2012; 46(2):241–52.

[31] Lê Cao K-A, Boitard S, Besse P. Sparse PLS discriminant analysis: biologically relevant feature selection and graphical displays for multiclass problems. BMC bioinformatics. 2011; 12(1):253. 21693065 10.1186/1471-2105-12-253 PMC3133555

[32] Jobson J. Multiple linear regression Applied multivariate data analysis: Springer; 1991 p. 219–398.

[33] Efroymson M. Multiple regression analysis. Mathematical methods for digital computers. 1960:191–203.

[34] Hutcheson GD. Ordinary least-squares regression. L Moutinho and GD Hutcheson, The SAGE dictionary of quantitative management research 2011:224–8.

[35] Ergon R, Granato D, Ares G. Principal component regression (PCR) and partial least squares regression (PLSR). Mathematical and statistical methods in food science and technology Wiley Blackwell, Chichester 2014:121–42.

[36] Tobias RD, editor An introduction to partial least squares regression. Proceedings of the twentieth annual SAS users group international conference; 1995: SAS Institute Inc Cary.

[37] Jamshidi S, Yadollahi A, Arab MM, Soltani M, Eftekhari M, Sabzalipoor H, et al Combining gene expression programming and genetic algorithm as a powerful hybrid modeling approach for pear rootstocks tissue culture media formulation. Plant Methods. 2019; 15(1):136. 31832078 10.1186/s13007-019-0520-y PMC6859635

[38] Jamshidi S, Yadollahi A, Ahmadi H, Arab M, Eftekhari M. Predicting in vitro culture medium macro-nutrients composition for pear rootstocks using regression analysis and neural network models. Frontiers in plant science. 2016; 7:274 10.3389/fpls.2016.00274 27066013 PMC4809900

[39] Sharma V, Rudnick DR, Irmak S. Development and evaluation of ordinary least squares regression models for predicting irrigated and rainfed maize and soybean yields. Transactions of the ASABE. 2013; 56(4):1361–78.

[40] Juhos K, Szabó S, Ladányi M. Influence of soil properties on crop yield: a multivariate statistical approach. International Agrophysics. 2015; 29(4). 10.1515/intag-2015-0057 27099408 PMC4834993

[41] Tzanakakis VA, Mauromoustakos A, Angelakis AN. Prediction of biomass production and nutrient uptake in land application using partial least squares regression analysis. Water. 2015;7(1):1–11.

[42] Nezami-Alanagh E, Garoosi G-A, Landín M, Gallego PP. Combining DOE with neurofuzzy logic for healthy mineral nutrition of pistachio rootstocks in vitro culture. Frontiers in Plant Science. 2018; 9:1474 10.3389/fpls.2018.01474 30374362 PMC6196285

[43] Ahmadi H, Golian A. Response surface and neural network models for performance of broiler chicks fed diets varying in digestible protein and critical amino acids from 11 to 17 days of age. Poultry science. 2011; 90(9):2085–96. 10.3382/ps.2011-01367 21844277

[44] Gago J, Martínez-Núñez L, Landín M, Gallego P. Artificial neural networks as an alternative to the traditional statistical methodology in plant research. Journal of plant physiology. 2010; 167(1):23–7. 10.1016/j.jplph.2009.07.007 19716625

[45] Agatonovic-Kustrin S, Beresford R. Basic concepts of artificial neural network (ANN) modeling and its application in pharmaceutical research. Journal of pharmaceutical and biomedical analysis. 2000; 22(5):717–27. 10.1016/s0731-7085(99)00272-1 10815714

[46] Patnaik P. Applications of neural networks to recovery of biological products. Biotechnology advances. 1999; 17(6):477–88. 10.1016/s0734-9750(99)00013-0 14538125

[47] Vassilopoulos AP, Bedi R. Adaptive neuro-fuzzy inference system in modelling fatigue life of multidirectional composite laminates. Computational Materials Science. 2008; 43(4):1086–93.

[48] Afriyie Mensah R, Xiao J, Das O, Jiang L, Xu Q, Alhassan MO. Application of Adaptive Neuro-Fuzzy Inference System in Flammability Parameter Prediction. Polymers. 2020; 12(1):122. 10.3390/polym12010122 PMC7022455 31948059

[49] Hesami M, Naderi R, Tohidfar M, Yoosefzadeh-Najafabadi M. Application of adaptive neuro-fuzzy inference system-non-dominated sorting genetic Algorithm-II (ANFIS-NSGAII) for modeling and optimizing somatic embryogenesis of Chrysanthemum. Frontiers in plant science. 2019; 10:869 10.3389/fpls.2019.00869 31333705 PMC6624437

[50] Hosseini M, Agereh SR, Khaledian Y, Zoghalchali HJ, Brevik EC, Naeini SARM. Comparison of multiple statistical techniques to predict soil phosphorus. Applied Soil Ecology. 2017; 114:123–31.

[51] Sheikhi A, Mirdehghan SH, Arab MM, Eftekhari M, Ahmadi H, Jamshidi S, et al Novel organic-based postharvest sanitizer formulation using Box Behnken design and mathematical modeling approach: A case study of fresh pistachio storage under modified atmosphere packaging. Postharvest Biology and Technology. 2020; 160:111047.

[52] Hesami M, Naderi R, Tohidfar M. Modeling and optimizing in vitro sterilization of Chrysanthemum via multilayer perceptron-non-dominated sorting genetic algorithm-II (MLP-NSGAII). Frontiers in plant science. 2019; 10. 10.3389/fpls.2019.00282 PMC6426794 30923529

[53] Hesami M, Naderi R, Tohidfar M. Modeling and optimizing medium composition for shoot regeneration of Chrysanthemum via radial basis function-non-dominated sorting genetic algorithm-II (RBF-NSGAII). Scientific Reports. 2019; 9(1):1–11. 10.1038/s41598-018-37186-2 31796784 PMC6890634

[54] Arab MM, Yadollahi A, Eftekhari M, Ahmadi H, Akbari M, Khorami SS. Modeling and optimizing a new culture medium for in vitro rooting of G× N15 Prunus rootstock using artificial neural network-genetic algorithm. Scientific reports. 2018; 8(1):1–18. 10.1038/s41598-017-17765-5 29967468 PMC6028477

[55] Zhang Q, Deng D, Dai W, Li J, Jin X. Optimization of culture conditions for differentiation of melon based on artificial neural network and genetic algorithm. Scientific Reports. 2020; 10(1):1–8. 10.1038/s41598-019-56847-4 32103071 PMC7044330

[56] Holland JH: Adaptation in natural and artificial systems: an introductory analysis with applications to biology, control, and artificial intelligence: MIT press; 1992.

[57] Shao Q, Rowe RC, York P. Comparison of neurofuzzy logic and neural networks in modelling experimental data of an immediate release tablet formulation. European journal of pharmaceutical sciences. 2006; 28(5):394–404. 10.1016/j.ejps.2006.04.007 16781126

[58] Jang J-S. ANFIS: adaptive-network-based fuzzy inference system. IEEE transactions on systems, man, and cybernetics. 1993; 23(3):665–85.

[59] Matlab V: 7.10. 0 (R2010a). The MathWorks Inc, Natick, Massachusetts 2010.

[60] XLSTAT, X. (2017). Data analysis and statistical solution for Microsoft Excel.

[61] GraphPad Prism 5 (2005) GraphPad Prism 5. GraphPad Software Inc., San Diego.

[62] Osama K, Mishra BN, Somvanshi P. Machine learning techniques in plant biology Plant Omics: The Omics of plant science: Springer; 2015 p. 731–54.

