# Supplementary material for: Modeling of paclitaxel biosynthesis elicitation in Corylus avellana cell culture using adaptive neuro-fuzzy inference system-genetic algorithm (ANFIS-GA) and multiple regression methods
Source: PLoS One. 2020 Aug 27;15(8):e0237478. doi: 10.1371/journal.pone.0237478 (PMC7451515; doi:10.1371/journal.pone.0237478)
Supplement: S1 Table — (DOCX) [file pone.0237478.s001.docx]

| **S1 Table.** Levels of input variables related to paclitaxel biosynthesis in *Corylus avellana* cell culture responding to cell extract (CE) and methyl-β-cyclodextrin (MBCD). | | | | | |
| --- | --- | --- | --- | --- | --- |
| Sample | CE concentration (% v/v) | MBCD concentration (mM) | CE adding day | Harvesting day | Paclitaxel  (µg l^-1^) |
| 1 | 0 | 0 | 13 | 15 | 24.220 ± 0.93 |
| 2 | 0 | 0 | 13 | 17 | 33.664 ± 0.09 |
| 3 | 0 | 0 | 13 | 19 | 41.230 ± 4.26 |
| 4 | 0 | 0 | 13 | 21 | 67.267 ± 5.85 |
| 5 | 0 | 0 | 13 | 23 | 37.053 ± 2.80 |
| 6 | 0 | 0 | 17 | 19 | 38.998 ± 1.51 |
| 7 | 0 | 0 | 17 | 21 | 70.266 ± 1.58 |
| 8 | 0 | 0 | 17 | 23 | 38.536 ± 1.66 |
| 9 | 0 | 50 | 13 | 15 | 52.062 ± 1.10 |
| 10 | 0 | 50 | 13 | 17 | 68.982 ± 1.78 |
| 11 | 0 | 50 | 13 | 19 | 77.484 ± 2.33 |
| 12 | 0 | 50 | 13 | 21 | 110.939 ± 2.30 |
| 13 | 0 | 50 | 13 | 23 | 108.829 ± 9.51 |
| 14 | 0 | 50 | 17 | 19 | 73.081 ± 1.07 |
| 15 | 0 | 50 | 17 | 21 | 108.448 ± 1.22 |
| 16 | 0 | 50 | 17 | 23 | 103.036 ± 5.65 |
| 17 | 1 | 0 | 13 | 15 | 24.882 ± 2.11 |
| 18 | 1 | 0 | 13 | 17 | 35.587 ± 2.29 |
| 19 | 1 | 0 | 13 | 19 | 45.458 ± 4.85 |
| 20 | 1 | 0 | 13 | 21 | 73.700 ± 10.11 |
| 21 | 1 | 0 | 13 | 23 | 43.538 ± 4.66 |
| 22 | 1 | 0 | 17 | 19 | 44.791 ± 4.22 |
| 23 | 1 | 0 | 17 | 21 | 75.368 ± 8.18 |
| 24 | 1 | 0 | 17 | 23 | 43.538 ± 4.66 |
| 25 | 1 | 50 | 13 | 15 | 54.549 ± 4.21 |
| 26 | 1 | 50 | 13 | 17 | 69.587 ± 5.15 |
| 27 | 1 | 50 | 13 | 19 | 78.924 ± 5.30 |
| 28 | 1 | 50 | 13 | 21 | 111.886 ± 10.30 |
| 29 | 1 | 50 | 13 | 23 | 98.538 ± 9.40 |
| 30 | 1 | 50 | 17 | 19 | 70.124 ± 6.82 |
| 31 | 1 | 50 | 17 | 21 | 109.293 ± 11.29 |
| 32 | 1 | 50 | 17 | 23 | 97.871 ± 13.32 |
| 33 | 2.5 | 0 | 13 | 15 | 65.995 ± 5.05 |
| 34 | 2.5 | 0 | 13 | 17 | 84.142 ± 8.09 |
| 35 | 2.5 | 0 | 13 | 19 | 94.021 ± 11.32 |
| 36 | 2.5 | 0 | 13 | 21 | 126.241 ± 9.08 |
| 37 | 2.5 | 0 | 13 | 23 | 89.604 ± 7.63 |
| 38 | 2.5 | 0 | 17 | 19 | 116.687 ± 7.96 |
| 39 | 2.5 | 0 | 17 | 21 | 158.140 ± 11.26 |
| 40 | 2.5 | 0 | 17 | 23 | 98.271 ± 8.40 |
| 41 | 2.5 | 50 | 13 | 15 | 117.995 ± 13.49 |
| 42 | 2.5 | 50 | 13 | 17 | 138.842 ± 9.48 |
| 43 | 2.5 | 50 | 13 | 19 | 151.354 ± 11.73 |
| 44 | 2.5 | 50 | 13 | 21 | 185.382 ± 8.94 |
| 45 | 2.5 | 50 | 13 | 23 | 189.938 ± 14.44 |
| 46 | 2.5 | 50 | 17 | 19 | 71.354 ± 8.90 |
| 47 | 2.5 | 50 | 17 | 21 | 117.296 ± 11.25 |
| 48 | 2.5 | 50 | 17 | 23 | 103.938 ± 7.55 |
| 49 | 5 | 0 | 13 | 15 | 81.153 ± 5.61 |
| 50 | 5 | 0 | 13 | 17 | 96.579 ± 3.41 |
| 51 | 5 | 0 | 13 | 19 | 109.868 ± 8.69 |
| 52 | 5 | 0 | 13 | 21 | 147.709 ± 8.79 |
| 53 | 5 | 0 | 13 | 23 | 112.029 ± 8.49 |
| 54 | 5 | 0 | 17 | 19 | 216.535 ± 12.14 |
| 55 | 5 | 0 | 17 | 21 | 266.921 ± 16.82 |
| 56 | 5 | 0 | 17 | 23 | 196.029 ± 14.98 |
| 57 | 5 | 50 | 13 | 15 | 140.487 ± 12.57 |
| 58 | 5 | 50 | 13 | 17 | 160.579 ± 12.46 |
| 59 | 5 | 50 | 13 | 19 | 175.868 ± 14.88 |
| 60 | 5 | 50 | 13 | 21 | 214.489 ± 16.75 |
| 61 | 5 | 50 | 13 | 23 | 207.696 ± 23.86 |
| 62 | 5 | 50 | 17 | 19 | 75.388 ± 5.82 |
| 63 | 5 | 50 | 17 | 21 | 113.147 ± 10.18 |
| 64 | 5 | 50 | 17 | 23 | 97.132 ± 7.13 |
| 65 | 10 | 0 | 13 | 15 | 36.549 ± 3.51 |
| 66 | 10 | 0 | 13 | 17 | 44.253 ± 3.35 |
| 67 | 10 | 0 | 13 | 19 | 55.124 ± 4.55 |
| 68 | 10 | 0 | 13 | 21 | 88.280 ± 6.87 |
| 69 | 10 | 0 | 13 | 23 | 54.205 ± 6.10 |
| 70 | 10 | 0 | 17 | 19 | 62.124 ± 4.08 |
| 71 | 10 | 0 | 17 | 21 | 93.451 ± 11.59 |
| 72 | 10 | 0 | 17 | 23 | 62.205 ± 5.06 |
| 73 | 10 | 50 | 13 | 15 | 66.216 ± 7.50 |
| 74 | 10 | 50 | 13 | 17 | 86.253 ± 5.16 |
| 75 | 10 | 50 | 13 | 19 | 95.458 ± 3.82 |
| 76 | 10 | 50 | 13 | 21 | 134.630 ± 17.98 |
| 77 | 10 | 50 | 13 | 23 | 121.479 ± 10.66 |
| 78 | 10 | 50 | 17 | 19 | 78.791 ± 5.63 |
| 79 | 10 | 50 | 17 | 21 | 116.502 ± 9.55 |
| 80 | 10 | 50 | 17 | 23 | 95.538 ± 10.69 |
