# Supplementary material for: Modeling of paclitaxel biosynthesis elicitation in Corylus avellana cell culture using adaptive neuro-fuzzy inference system-genetic algorithm (ANFIS-GA) and multiple regression methods
Source: PLoS One. 2020 Aug 27;15(8):e0237478. doi: 10.1371/journal.pone.0237478 (PMC7451515; doi:10.1371/journal.pone.0237478)
Supplement: S2 Table — (DOCX) [file pone.0237478.s002.docx]

| **S2 Table.** Levels of input variables related to paclitaxel biosynthesis in *Corylus avellana* cell culture responding to culture filtrate (CF) and methyl-β-cyclodextrin (MBCD). | | | | | |
| --- | --- | --- | --- | --- | --- |
| Sample | CF concentration (% v/v) | MBCD concentration (mM) | CF adding day | Harvesting day | Paclitaxel  (µg l^-1^) |
| 1 | 0 | 0 | 13 | 15 | 24.220 ± 0.93 |
| 2 | 0 | 0 | 13 | 17 | 33.664 ± 0.09 |
| 3 | 0 | 0 | 13 | 19 | 41.230 ± 4.26 |
| 4 | 0 | 0 | 13 | 21 | 67.267 ± 5.85 |
| 5 | 0 | 0 | 13 | 23 | 37.053 ± 2.80 |
| 6 | 0 | 0 | 17 | 19 | 38.998 ± 1.51 |
| 7 | 0 | 0 | 17 | 21 | 70.266 ± 1.58 |
| 8 | 0 | 0 | 17 | 23 | 38.536 ± 1.66 |
| 9 | 0 | 50 | 13 | 15 | 52.062 ± 1.10 |
| 10 | 0 | 50 | 13 | 17 | 68.982 ± 1.78 |
| 11 | 0 | 50 | 13 | 19 | 77.484 ± 2.33 |
| 12 | 0 | 50 | 13 | 21 | 110.939 ± 2.30 |
| 13 | 0 | 50 | 13 | 23 | 108.829 ± 9.51 |
| 14 | 0 | 50 | 17 | 19 | 73.081 ± 1.07 |
| 15 | 0 | 50 | 17 | 21 | 108.448 ± 1.22 |
| 16 | 0 | 50 | 17 | 23 | 103.036 ± 5.65 |
| 17 | 1 | 0 | 13 | 15 | 25.882 ± 1.77 |
| 18 | 1 | 0 | 13 | 17 | 35.920 ± 3.13 |
| 19 | 1 | 0 | 13 | 19 | 43.258 ± 4.02 |
| 20 | 1 | 0 | 13 | 21 | 73.165 ± 8.46 |
| 21 | 1 | 0 | 13 | 23 | 41.205 ± 3.80 |
| 22 | 1 | 0 | 17 | 19 | 42.124 ± 1.62 |
| 23 | 1 | 0 | 17 | 21 | 73.586 ± 3.91 |
| 24 | 1 | 0 | 17 | 23 | 42.205 ± 3.82 |
| 25 | 1 | 50 | 13 | 15 | 54.549 ± 4.21 |
| 26 | 1 | 50 | 13 | 17 | 69.587 ± 5.15 |
| 27 | 1 | 50 | 13 | 19 | 78.924 ± 5.30 |
| 28 | 1 | 50 | 13 | 21 | 111.886 ± 10.30 |
| 29 | 1 | 50 | 13 | 23 | 98.538 ± 9.40 |
| 30 | 1 | 50 | 17 | 19 | 68.124 ± 6.20 |
| 31 | 1 | 50 | 17 | 21 | 107.538 ± 8.62 |
| 32 | 1 | 50 | 17 | 23 | 98.871 ± 10.30 |
| 33 | 2.5 | 0 | 13 | 15 | 26.328 ± 2.72 |
| 34 | 2.5 | 0 | 13 | 17 | 37.476 ± 2.55 |
| 35 | 2.5 | 0 | 13 | 19 | 44.354 ± 3.39 |
| 36 | 2.5 | 0 | 13 | 21 | 77.857 ± 7.06 |
| 37 | 2.5 | 0 | 13 | 23 | 44.604 ± 4.50 |
| 38 | 2.5 | 0 | 17 | 19 | 41.021 ± 3.22 |
| 39 | 2.5 | 0 | 17 | 21 | 78.547 ± 3.86 |
| 40 | 2.5 | 0 | 17 | 23 | 46.938 ± 2.59 |
| 41 | 2.5 | 50 | 13 | 15 | 117.995 ± 13.49 |
| 42 | 2.5 | 50 | 13 | 17 | 138.842 ± 9.48 |
| 43 | 2.5 | 50 | 13 | 19 | 151.354 ± 11.73 |
| 44 | 2.5 | 50 | 13 | 21 | 185.382 ± 8.94 |
| 45 | 2.5 | 50 | 13 | 23 | 189.938 ± 14.44 |
| 46 | 2.5 | 50 | 17 | 19 | 76.354 ± 6.30 |
| 47 | 2.5 | 50 | 17 | 21 | 114.849 ± 7.27 |
| 48 | 2.5 | 50 | 17 | 23 | 118.271 ± 11.85 |
| 49 | 5 | 0 | 13 | 15 | 24.153 ± 1.61 |
| 50 | 5 | 0 | 13 | 17 | 33.246 ± 2.51 |
| 51 | 5 | 0 | 13 | 19 | 40.535 ± 2.97 |
| 52 | 5 | 0 | 13 | 21 | 79.798 ± 4.74 |
| 53 | 5 | 0 | 13 | 23 | 46.029 ± 3.59 |
| 54 | 5 | 0 | 17 | 19 | 46.388 ± 2.84 |
| 55 | 5 | 0 | 17 | 21 | 81.595 ± 6.20 |
| 56 | 5 | 0 | 17 | 23 | 48.465 ± 1.18 |
| 57 | 5 | 50 | 13 | 15 | 140.487 ± 12.57 |
| 58 | 5 | 50 | 13 | 17 | 160.579 ± 12.46 |
| 59 | 5 | 50 | 13 | 19 | 175.868 ± 14.88 |
| 60 | 5 | 50 | 13 | 21 | 214.489 ± 16.75 |
| 61 | 5 | 50 | 13 | 23 | 207.696 ± 23.86 |
| 62 | 5 | 50 | 17 | 19 | 70.388 ± 5.82 |
| 63 | 5 | 50 | 17 | 21 | 118.077 ± 9.32 |
| 64 | 5 | 50 | 17 | 23 | 109.132 ± 10.70 |
| 65 | 10 | 0 | 13 | 15 | 29.549 ± 1.96 |
| 66 | 10 | 0 | 13 | 17 | 39.587 ± 1.90 |
| 67 | 10 | 0 | 13 | 19 | 47.124 ± 3.14 |
| 68 | 10 | 0 | 13 | 21 | 82.310 ± 6.22 |
| 69 | 10 | 0 | 13 | 23 | 46.538 ± 2.48 |
| 70 | 10 | 0 | 17 | 19 | 146.458 ± 12.05 |
| 71 | 10 | 0 | 17 | 21 | 187.117 ± 10.75 |
| 72 | 10 | 0 | 17 | 23 | 131.538 ± 9.42 |
| 73 | 10 | 50 | 13 | 15 | 66.216 ± 7.50 |
| 74 | 10 | 50 | 13 | 17 | 86.253 ± 5.16 |
| 75 | 10 | 50 | 13 | 19 | 95.458 ± 3.82 |
| 76 | 10 | 50 | 13 | 21 | 134.630 ± 17.98 |
| 77 | 10 | 50 | 13 | 23 | 121.479 ± 10.66 |
| 78 | 10 | 50 | 17 | 19 | 178.791 ± 15.96 |
| 79 | 10 | 50 | 17 | 21 | 241.921 ± 15.03 |
| 80 | 10 | 50 | 17 | 23 | 222.205 ± 28.31 |
