# Supplementary material for: Modeling of paclitaxel biosynthesis elicitation in Corylus avellana cell culture using adaptive neuro-fuzzy inference system-genetic algorithm (ANFIS-GA) and multiple regression methods
Source: PLoS One. 2020 Aug 27;15(8):e0237478. doi: 10.1371/journal.pone.0237478 (PMC7451515; doi:10.1371/journal.pone.0237478)
Supplement: S3 Table — (DOCX) [file pone.0237478.s003.docx]

| **S3 Table.** Levels of input variables related to paclitaxel biosynthesis in *Corylus avellana* cell culture responding to cell wall (CW) and methyl-β-cyclodextrin (MBCD). | | | | | |
| --- | --- | --- | --- | --- | --- |
| Sample | CW concentration (% v/v) | MBCD concentration (mM) | CW adding day | Harvesting day | Paclitaxel  (µg l^-1^) |
| 1 | 0 | 0 | 13 | 15 | 24.220 ± 0.93 |
| 2 | 0 | 0 | 13 | 17 | 33.664 ± 0.09 |
| 3 | 0 | 0 | 13 | 19 | 41.230 ± 4.26 |
| 4 | 0 | 0 | 13 | 21 | 67.267 ± 5.85 |
| 5 | 0 | 0 | 13 | 23 | 37.053 ± 2.80 |
| 6 | 0 | 0 | 17 | 19 | 38.998 ± 1.51 |
| 7 | 0 | 0 | 17 | 21 | 70.266 ± 1.58 |
| 8 | 0 | 0 | 17 | 23 | 38.536 ± 1.66 |
| 9 | 0 | 50 | 13 | 15 | 52.062 ± 1.10 |
| 10 | 0 | 50 | 13 | 17 | 68.982 ± 1.78 |
| 11 | 0 | 50 | 13 | 19 | 77.484 ± 2.33 |
| 12 | 0 | 50 | 13 | 21 | 110.939 ± 2.30 |
| 13 | 0 | 50 | 13 | 23 | 108.829 ± 9.51 |
| 14 | 0 | 50 | 17 | 19 | 73.081 ± 1.07 |
| 15 | 0 | 50 | 17 | 21 | 108.448 ± 1.22 |
| 16 | 0 | 50 | 17 | 23 | 103.036 ± 5.65 |
| 17 | 1 | 0 | 13 | 15 | 23.216 ± 0.93 |
| 18 | 1 | 0 | 13 | 17 | 31.920 ± 2.26 |
| 19 | 1 | 0 | 13 | 19 | 41.591 ± 2.82 |
| 20 | 1 | 0 | 13 | 21 | 75.803 ± 6.18 |
| 21 | 1 | 0 | 13 | 23 | 42.871 ± 3.70 |
| 22 | 1 | 0 | 17 | 19 | 40.124 ± 2.29 |
| 23 | 1 | 0 | 17 | 21 | 71.820 ± 6.70 |
| 24 | 1 | 0 | 17 | 23 | 42.205 ± 3.82 |
| 25 | 1 | 50 | 13 | 15 | 46.216 ± 3.89 |
| 26 | 1 | 50 | 13 | 17 | 66.920 ± 3.99 |
| 27 | 1 | 50 | 13 | 19 | 76.591 ± 5.83 |
| 28 | 1 | 50 | 13 | 21 | 113.389 ± 11.12 |
| 29 | 1 | 50 | 13 | 23 | 100.538 ± 9.62 |
| 30 | 1 | 50 | 17 | 19 | 71.124 ± 6.44 |
| 31 | 1 | 50 | 17 | 21 | 110.773 ± 8.02 |
| 32 | 1 | 50 | 17 | 23 | 95.871 ± 10.66 |
| 33 | 2.5 | 0 | 13 | 15 | 57.995 ± 5.65 |
| 34 | 2.5 | 0 | 13 | 17 | 73.809 ± 5.65 |
| 35 | 2.5 | 0 | 13 | 19 | 82.354 ± 7.06 |
| 36 | 2.5 | 0 | 13 | 21 | 121.399 ± 9.20 |
| 37 | 2.5 | 0 | 13 | 23 | 81.271 ± 6.92 |
| 38 | 2.5 | 0 | 17 | 19 | 205.021 ± 19.12 |
| 39 | 2.5 | 0 | 17 | 21 | 245.004 ± 15.71 |
| 40 | 2.5 | 0 | 17 | 23 | 170.271 ± 15.58 |
| 41 | 2.5 | 50 | 13 | 15 | 84.995 ± 13.31 |
| 42 | 2.5 | 50 | 13 | 17 | 105.509 ± 8.85 |
| 43 | 2.5 | 50 | 13 | 19 | 116.687 ± 6.26 |
| 44 | 2.5 | 50 | 13 | 21 | 156.460 ± 11.23 |
| 45 | 2.5 | 50 | 13 | 23 | 141.938 ± 10.51 |
| 46 | 2.5 | 50 | 17 | 19 | 327.687 ± 23.50 |
| 47 | 2.5 | 50 | 17 | 21 | 402.452 ± 22.44 |
| 48 | 2.5 | 50 | 17 | 23 | 382.604 ± 19.97 |
| 49 | 5 | 0 | 13 | 15 | 58.487 ± 3.44 |
| 50 | 5 | 0 | 13 | 17 | 78.246 ± 5.45 |
| 51 | 5 | 0 | 13 | 19 | 85.868 ± 6.12 |
| 52 | 5 | 0 | 13 | 21 | 131.339 ± 12.10 |
| 53 | 5 | 0 | 13 | 23 | 86.029 ± 5.01 |
| 54 | 5 | 0 | 17 | 19 | 200.388 ± 15.47 |
| 55 | 5 | 0 | 17 | 21 | 255.421 ± 17.23 |
| 56 | 5 | 0 | 17 | 23 | 178.132 ± 15.15 |
| 57 | 5 | 50 | 13 | 15 | 92.487 ± 7.57 |
| 58 | 5 | 50 | 13 | 17 | 113.579 ± 7.24 |
| 59 | 5 | 50 | 13 | 19 | 131.868 ± 15.68 |
| 60 | 5 | 50 | 13 | 21 | 174.828 ± 12.58 |
| 61 | 5 | 50 | 13 | 23 | 162.029 ± 15.25 |
| 62 | 5 | 50 | 17 | 19 | 307.055 ± 17.79 |
| 63 | 5 | 50 | 17 | 21 | 371.883 ± 26.97 |
| 64 | 5 | 50 | 17 | 23 | 362.465 ± 19.17 |
| 65 | 10 | 0 | 13 | 15 | 18.549 ± 2.27 |
| 66 | 10 | 0 | 13 | 17 | 23.920 ± 2.74 |
| 67 | 10 | 0 | 13 | 19 | 30.458 ± 2.18 |
| 68 | 10 | 0 | 13 | 21 | 59.639 ± 4.30 |
| 69 | 10 | 0 | 13 | 23 | 28.538 ± 2.47 |
| 70 | 10 | 0 | 17 | 19 | 61.791 ± 4.70 |
| 71 | 10 | 0 | 17 | 21 | 96.446 ± 10.84 |
| 72 | 10 | 0 | 17 | 23 | 63.205 ± 5.75 |
| 73 | 10 | 50 | 13 | 15 | 46.216 ± 5.21 |
| 74 | 10 | 50 | 13 | 17 | 56.253 ± 5.16 |
| 75 | 10 | 50 | 13 | 19 | 95.458 ± 3.82 |
| 76 | 10 | 50 | 13 | 21 | 95.135 ± 11.48 |
| 77 | 10 | 50 | 13 | 23 | 91.479 ± 10.66 |
| 78 | 10 | 50 | 17 | 19 | 102.791 ± 12.00 |
| 79 | 10 | 50 | 17 | 21 | 145.102 ± 8.58 |
| 80 | 10 | 50 | 17 | 23 | 130.538 ± 11.03 |
